# Management of penile post-circumcision ischemia by pentoxifylline infusion and hyperbaric oxygen therapy

**DOI:** 10.1186/s12894-023-01284-9

**Published:** 2023-07-12

**Authors:** Mohamed A Baky Fahmy, Tarek Abdelazeem Sabra, Sarah Magdy Abdelmohsen

**Affiliations:** 1 Faculty of Medicine, Pediatric Surgery Department, Al-Azhar University, Cairo, Egypt; 2 Pediatric Surgery Department, Assiut University Hospital, Assiut, Egypt; 3 Lecturer of Pediatric Surgery, Faculty of Medicine, Pediatric Surgery Unit, Aswan University, Aswan, Egypt

**Keywords:** Male circumcision, Glans necrosis, Penile Ischemia, Monopolar diathermy, Hyperbaric oxygen, Pentoxifylline

## Abstract

**Background:**

Post-circumcision penile ischemia is a devastating complication. We will present our experience in managing children with various forms of penile ischemia.

**Materials and methods:**

This cohort prospective observational and interventional study was performed on all male children with post-circumcision penile ischemia between April 2017 and October 2021. A designed and approved protocol includes a combination of early pentoxifylline infusion, hyperbaric oxygen inhalation, early catheterization, and appropriate surgical debridement were applied for patients with deep ischemia 11/23, mainly the necrotic skin and subcutaneous tissues. Data of patient age, anesthesia method, monopolar diathermy usage, early presentation and positive wound culture were collected and analyzed statistically.

**Results:**

During the study period 3,382 children were circumcised for non-medical reasons; 23 children were diagnosed with penile ischemia (0.7%), among other complications (9%). Most of the penile ischemia is associated with the use of monopolar diathermy (74%). The use of compressive wound dressing to control post-circumcision bleeding and infections is also responsible for ischemia in 52.2% and 43.5% of the cases. Inexperienced physicians were commonly responsible for ischemia (73.9%). Patients managed at first 24 h had better outcomes than those who were presented later (p = 0.001).

**Conclusion:**

In children with post-circumcision penile ischemia, a combination of hyperbaric oxygen therapy and pentoxifylline is especially effective for patients with skin and facial necrosis, this management reduces penile tissue loss.

## Introduction

Due to traditional and religious beliefs, male circumcision (MC) is still the most common surgical practice in Egypt and some other countries. The prevalence of MC in Egypt is approximately 94.7% [1]. The incidence of MC-related complications is variable and reported in about 1.5–5% [2]. These complications have a negative impaction on the sexual life and overall quality of life of the patients concerned, in addition to the besmirch that will follow the physicians responsible for these complications. Early minor complications such as bleeding, inconvenient skin excision, and surgical area infection are common. Early severe complications include glandular amputation, urinary retention, urethral injuries, and penile necrosis. Delayed complications include adhesions, skin bridges, epidermal inclusion cysts, phimosis, chordee, trapped penis, urethrocutaneous fistula, and meatal strictures [3, 4&^5^]. The most common complication of conventional circumcision are edema (37.6%) then bleeding (2.9%) [6]. Infection is the third most common complication (2.5%) [6&^7^]. Penile ischemia is the most serious complication; which may involves the skin and underlying fascia and this could be recoverable, but deep ischemia involving the glans penis and/or the erectile tissue, may end with a partial or complete penile loss and it will be associated with psychological trauma and later on it will impact on the patient’s life [5]. In literature different methods and pharmacologic agents have been reported for the management of penile ischemia; like enoxaparin, iloprost, corticosteroids, unfractionated heparin sodium, alteplase and caudal anesthesia [8,9]. All those modalities of treatment have either a limited success rate or were applied for a limited number of cases [8]; however, there is no currently available study about the treatment of choice [10]. Based on our basic information about HBOT and pentoxifylline as potent vasodilators and other agents that increase erythrocyte flexibility, reduce blood viscosity, and increase microcirculatory flow and tissue perfusion [11], we came up with a protocol for the management of children with post-circumcision penile ischemia by improving tissue revascularization, oxygenation, and infection control. HBOT in children means inhalation of 100% oxygen at pressures of up to 760 mmHg, which is used to increase the amount of oxygen dissolved in plasma from 3 mL/L at sea level to 60 mL/L, which is the concentration required for normal tissue perfusion [10]. Also this level of plasma oxygen can reach the obstructed areas of resting ischemic tissues, where red blood cells (RBCs) cannot reach and without the need for oxygen carried by the hemoglobin [10,11].

### Patients and methods

In this cohort prospective observational and interventional study, all male children who had penile ischemia after circumcision were included from April 2017 to October 2021. A full medical history, timing and method of circumcision, degree of penile ischemia were assessed roughly on clinical bases. Ischemia with any penile tissue loss was considered as a deep ischemia, but the only color changes with preservation of penile contour and structures were considered as a superficial one. There is no history of any penile abnormalities, bleeding disorders, episodes of priapism, or history medications in any of the included cases. A specially designed protocol includes an early admission of such children to the pediatric intensive care unit and urethral catheterization using a suitable-size age-appropriate silicon Foley’s catheter. Wound swabs and blood samples were taken initially from all patients for culture and sensitivity tests.

Intravenous pentoxifylline infusion was started at a dose of 10 mg/kg/day divided into three equal doses for 5–7 days with good monitoring of its main side effects, of tachycardia and convulsions. An IV third generation cephalosporin antibiotic (Cefotaxime sodium) with suitable doses (according to age and child’s body weight) was started for all patients; then, the treatment was mandated to the suitable susceptible antibiotics according to the culture and sensitivity results. This protocol was started and maintained during the hospital stay. HBOT was carried out in a specially designed cabinet for infants and neonates; it looks like a large incubator with monitoring system to measure the oxygen tension, pulse oximeter and a screen displaying a video games. The barometric pressures of up to 760 mmHg for 30 min daily for 7 days was started, then the duration was gradually increased to one hour daily until the discharge of the patient at 10th to 21st days. Children who had been diagnosed to have an upper respiratory tract infection were excluded. An audiogram was done for all children before the commitment of HBOT and repeated to detect any diverse hearing changes. No one of our cases had a history of seizure disorders.

**Fig. 1:**
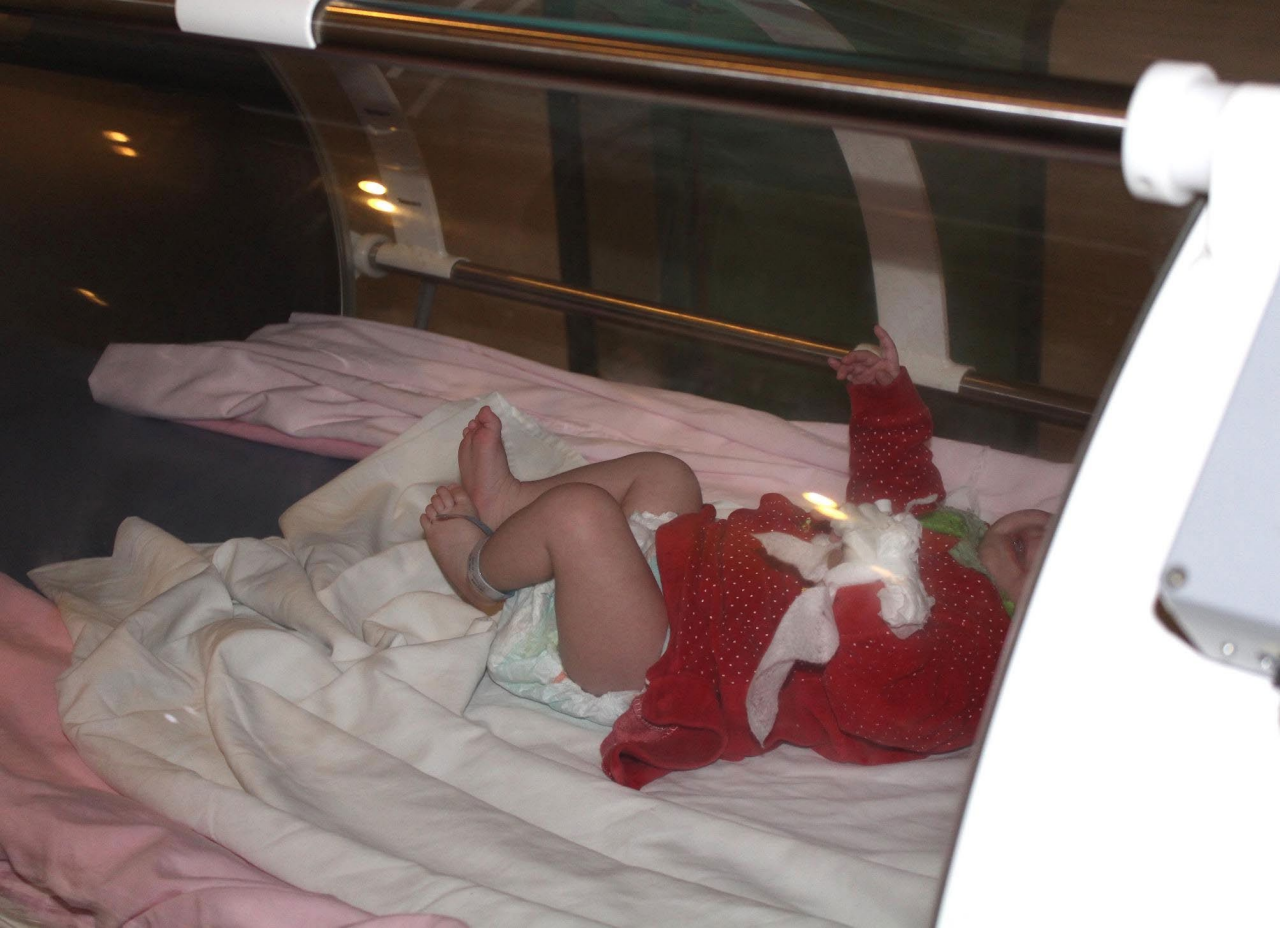
A special cabinet of hyperbaric oxygen was specially designed for infants and neonates

The HBO chamber (Fig. 1) was kept warm, and the younger babies were fed before entering the chamber and again if signs of hunger appreciated during therapy. The baby was continuously monitored with a pulse oximeter during the HBO sessions and infrequently in the inpatient department.

Older children were managed in the conventional HBO chamber with the same regimen. Prompt surgical debridement for the necrotic skin and underlying fascia was applied for eleven cases and repeated when required, but dressing with nitroglycerin ointment (2%) was done for all patients. Tissue considered necrotic when it looks blackish and detached from the surrounding or underlying structures. The treatment continued through the admission period. Patients discharged only when there is no general signs of infection, their wound healed with an evident epithelization and a culture swab from the affected tissue is negative for any pathological organism. The follow-up period ranged from 6 months to 3 years (mean 18 months). Some cases were individually scheduled for further surgical reconstruction according to the degree of tissue loss; and they were categorized to: - patients with only skin loss, patients with partial penile loss and patients with a complete penile ablation.

**Fig. 2:**
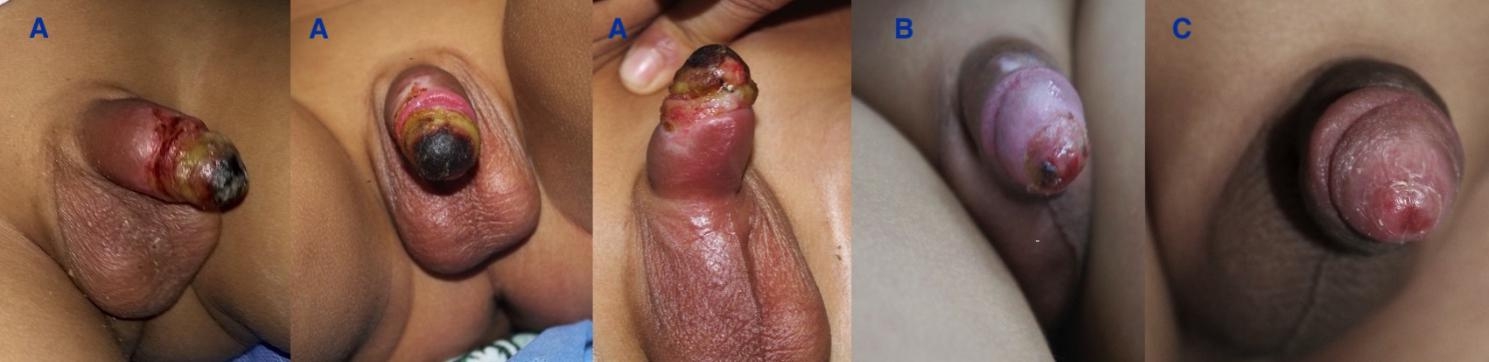
A 18 months old child presented with post-circumcision superficial glandular necrosis after guillotine circumcision with application of the monopolar diathermy to stop frenular bleeding and improved with the designed protocol (**A**: 1st presentation, **B**: 2 weeks after starting the treatment protocol and **C**: at l6 months follow-up)

**Fig. 3:**
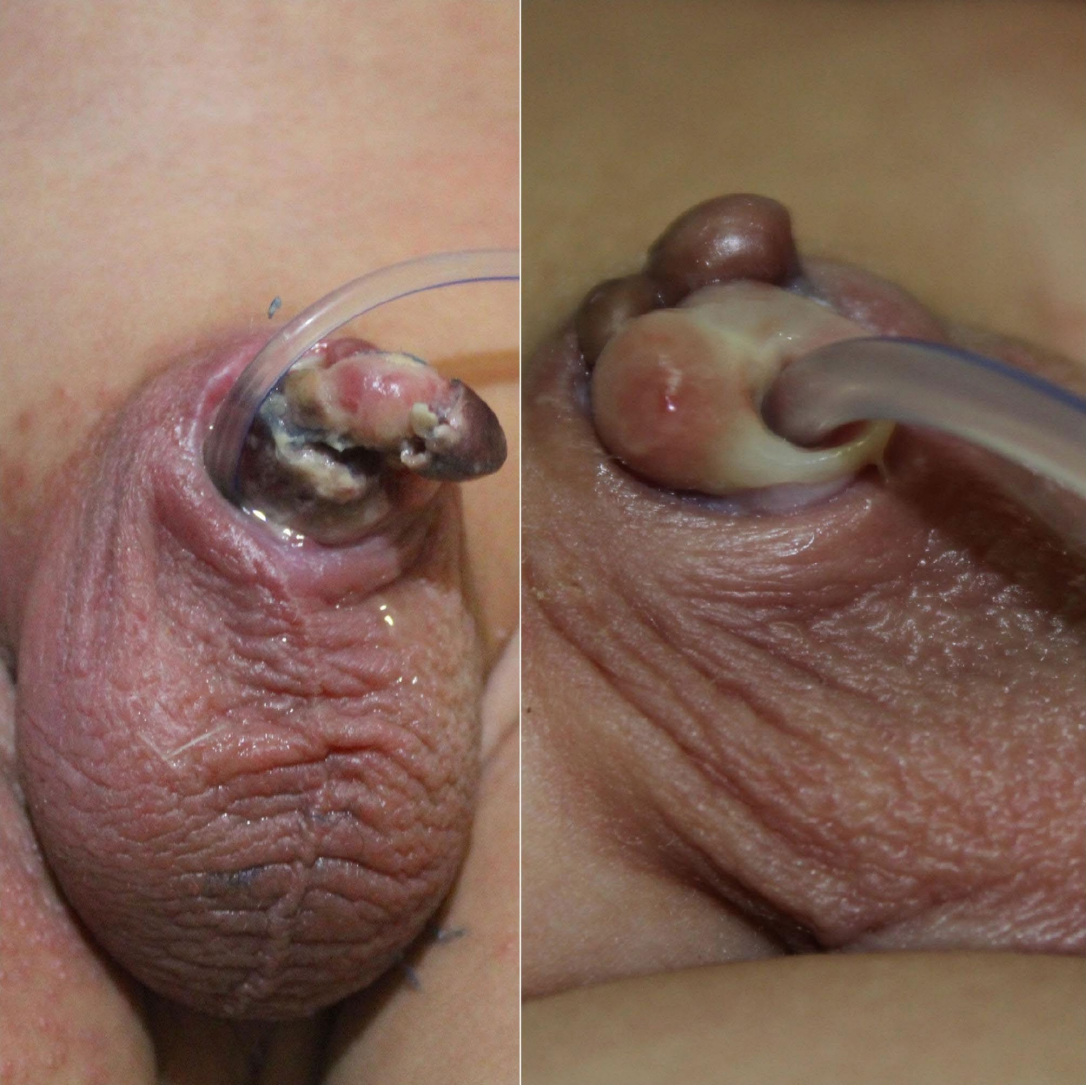
Six months old child with a superficial whole-penis ischemia with partial improvement, at one month he ended with a distal glandular and urethral loss

Data on patients’ ages, the level of experience of the circumcisor, the technique of MC, the kind of anesthesia used, any monopolar diathermy usage, wound suturing after MC, the duration between MC and presentation for our protocol were analyzed. Early detected patient is defined as a patient diagnosed and started the protocol within the first 24 h, the late-presenter are patient diagnosed after 24 h. Grade of ischemia and the patient’s final condition at discharge and after six months were collected and analyzed. The Grade of ischemia is defined as superficial glandular, superficial whole-penis, deep ischemia, gangerne, tissue loss, scrotal involvement and Fournier’s gangrene. Ischemia is a condition in which blood flow (and thus oxygen) is restricted or reduced in a part of the body. The superficial ischemia of the penis is the ischemia that extends up to the superficial dartos fascia, with preservation of penile tissue and contour. (Fig. 2), but deep penile ischemia is defined as penile ischemia extended beyond the superficial dartos fascia and it is diagnosed clinically if there is any tissue loss, or exposure of deep structures -mainly the corporal tissues- were detected (Fig. 3).

### Inclusion and exclusion criteria

Only male children aged one month up to 18 years old were included. Male children who had MC complications other than ischemia were excluded from the study.

Our university ethical committee (The institutional review board of the Faculty of Medicine Assiut University) approved this study. Also, parents and older children (from 16 to 18 years old) signed an informed consent for inclusion in the study and publishing their data and photos; with removal of any patient’s identity. All methods used in this study were performed in accordance with the relevant guidelines and regulations of the Declarations of Helsinki.

### Statistical analysis

SPSS version 20 was used for data analysis, categorical variables were presented as frequencies and proportions while continuous variables were presented as mean values and standard deviations. Pearson’s chi-squared test was used to evaluate differences between groups. P-values of < 0.05 were considered statistically significant.

## Results

Twenty-three male children with penile ischemia were referred among another 304 with another different complications, which were reported in 3382 circumcised children in the referring centers during the period between April 2017 and October 2021. The incidence of post-circumcision penile ischemia, among other complications, was 0.7%, and the overall complication rate was 9%. The Guillotine technique of circumcision was the most frequently technique used (85% of the circumcised cases), and plastibell technique was used for 10%, and dorsal slit technique in 5% of the 3382 circumcised children. Guillotine technique was associated with glans necrosis in 16 (69.6% of the penile ischemia), 6 (26%) with the dorsal slit technique and only in one patient (4.4%) of plastibell technique group. Monopolar cauterization was the most common risk factor for circumcision-related penile ischemia (74% of the cases), other patient had no history of either mono or bipolar diathermy application.

The experience level of personnel who performed the circumcision was the general practitioners and junior doctors in 14 (61%), specialists in 4 (17%) and unlicensed traditional practitioners in 5 (22%). Local anesthesia in the form of dorsal penile nerve block (DPNB) was associated with an increased risk of penile ischemia; 10 cases (44%), general anesthesia in 4 (17%) and MC without anesthesia in 9 (39%). Since the patients were referred to our center, we are unable to determine whether the local anesthetic agent used for DPNB contain a vasoconstrictor or not. Presence of surgical stitches was detected in 11 cases (48%) and history of compressive wound dressing after circumcision bleeding confirmed in 12 cases (52%). Post-circumcision infection diagnosed in 10 patients (44%), and the main organism isolated was Staphylococcus aureus (45%), followed by Staphylococcus epidermidis (30%), Streptococcus pyogenes (15%) and Actinobacteria Klebsiella (10%). Patients who had deep degrees of ischemia were associated with more anaerobic infection and had worse prognoses (p = 0.003). (Table 1)

**Table 1:** Percentage of the isolated organisms in cases of superficial or deep ischemia

Organism isolated	Staphylococcus aureus	Staphylococcus epidermidis	Streptococcus pyogenes	Actinobacteria Klebsiella
Superficial ischemia (%)	38%	24%	7%	2%
Deep ischemia (%)	7%	6%	8%	8%

Early detected cases (within the first 24 h), Late presented cases (more than 24 h).

Patients who were diagnosed and admitted early (9 patients; 39%) (within 24 h after MC) had better outcomes than those whose who were diagnosed late (14; 61%). Hence, the patients who had good outcomes were those with superficial degree of ischemia (p = 0.001) (Fig. 2). A good outcome means spontaneous resolution without tissue loss, while bad outcome means the patient ended with a significant penile tissue loss. (Table 2)

**Table 2:** The outcome of a penile ischemia according to the beginning of the management

The outcome	Time of diagnosis and admission		*P-value*
	Early admitted patients	Delayed admitted patients	
Good	8	4	0.001
**Bad**	**1**	**10**	**0.001**
**Total (23)**	**9 (39%)**	**14 (61%)**	

Pearson’s Chi-Square test, *P-value* less than 0.05 is considered statistically significant

Early detected cases (within the first 24 h), Late presented cases (more than 24 h)

**Fig. 4:**
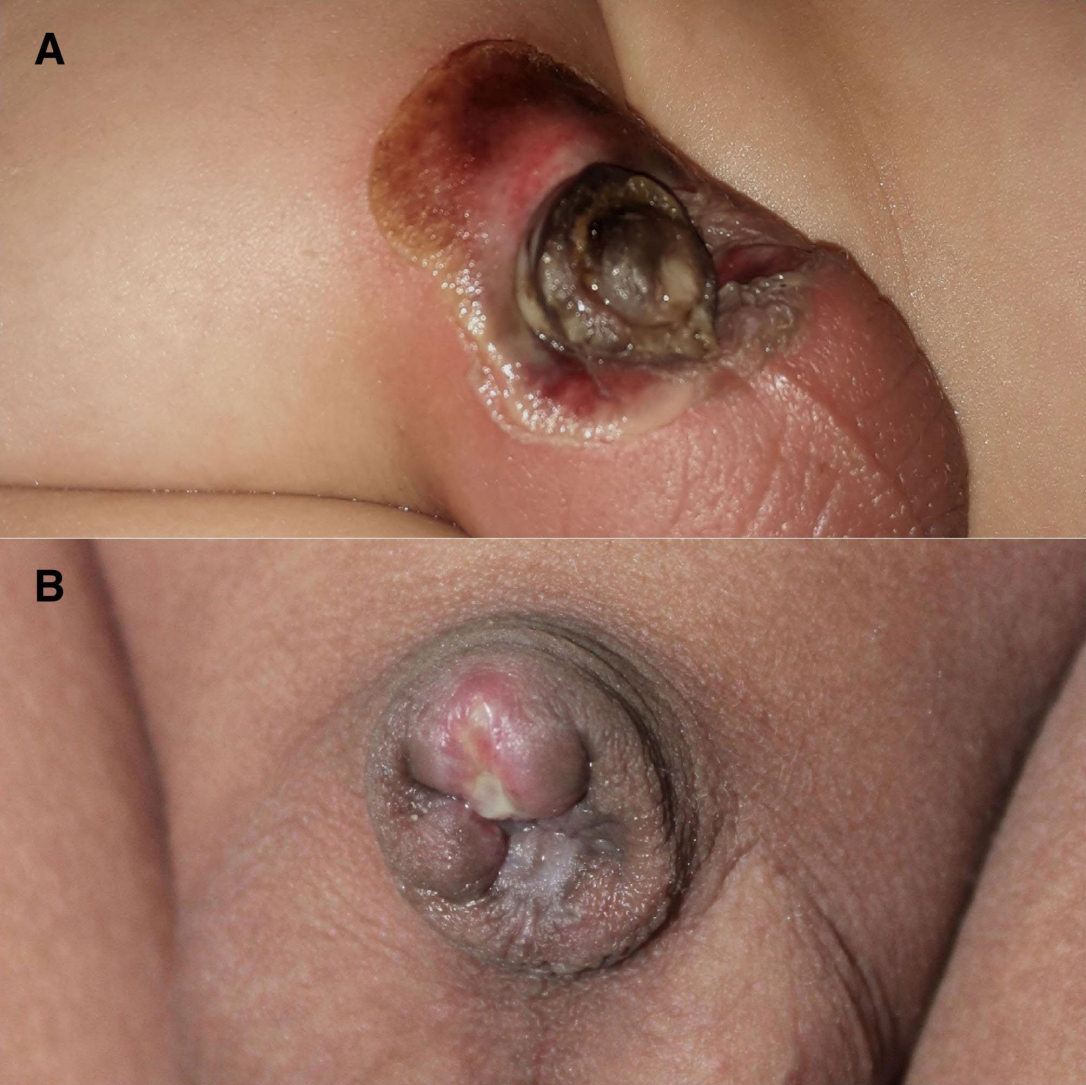
Four months old child presented with a superficial whole penile ischemia (**A**) with an acceptable recovery and minimal penile loss, 3months after treatment with our protocol (**B**)

**Fig. 5:**
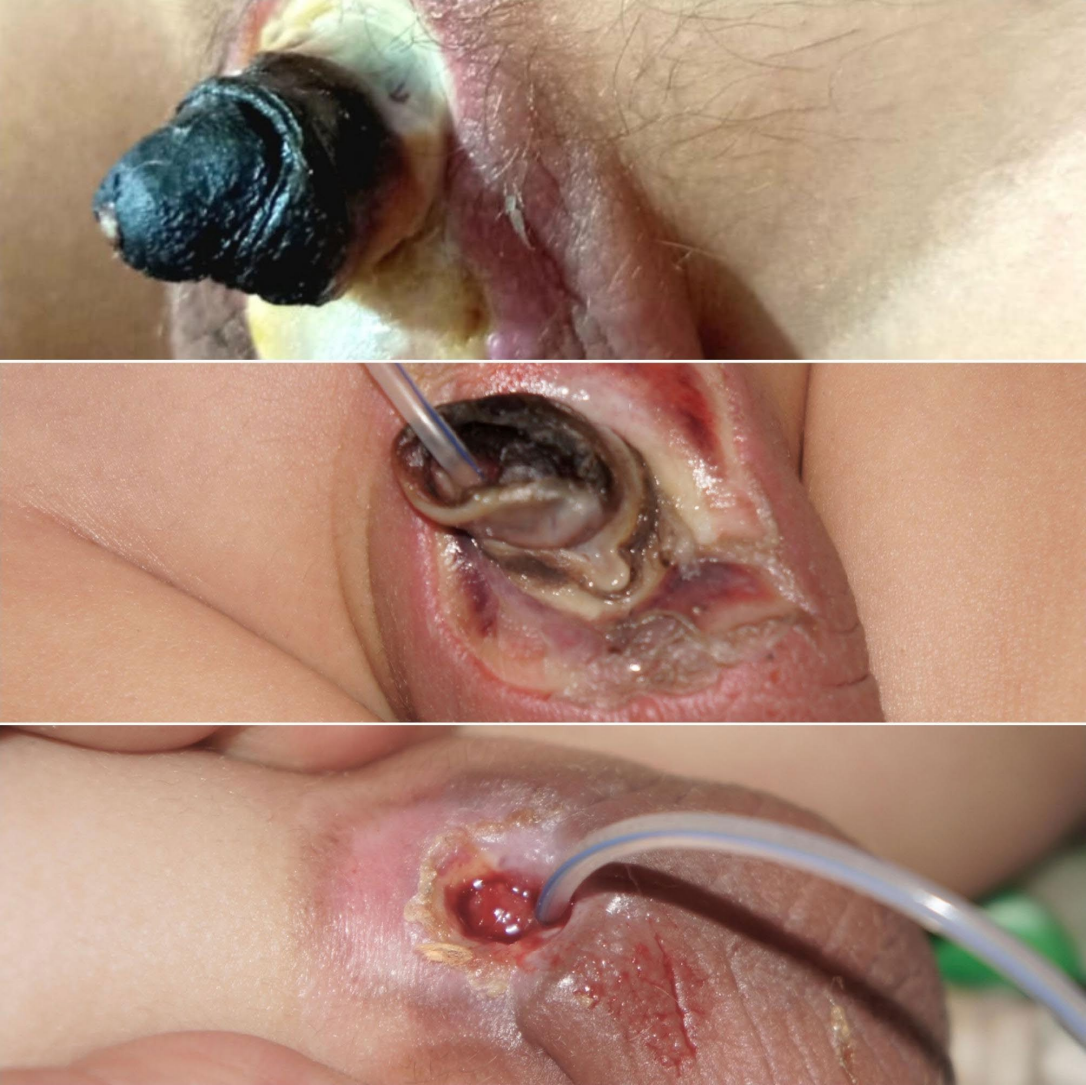
A one year-old boy with penile gangrene and ischemia around the base of the penis who experienced a partial improvement at the area around the base just six weeks after the injury, later on the child developed complete penile loss

The scale of the ischemia was a superficial glandular in 8 (34.8%), superficial ischemia involved the whole-penis in 4 (17.4%) (Fig. 3) Deep ischemia: 8 (35%) (Figs. 4 and 5), complete penile gangrene encountered in 1 (4.4%), tissue loss in 4 (17.4%) (Figs. 3, 4 and 5). Fournier’s gangrene was confirmed in 1(4.4%) (Fig. 5).

**Fig. 6:**
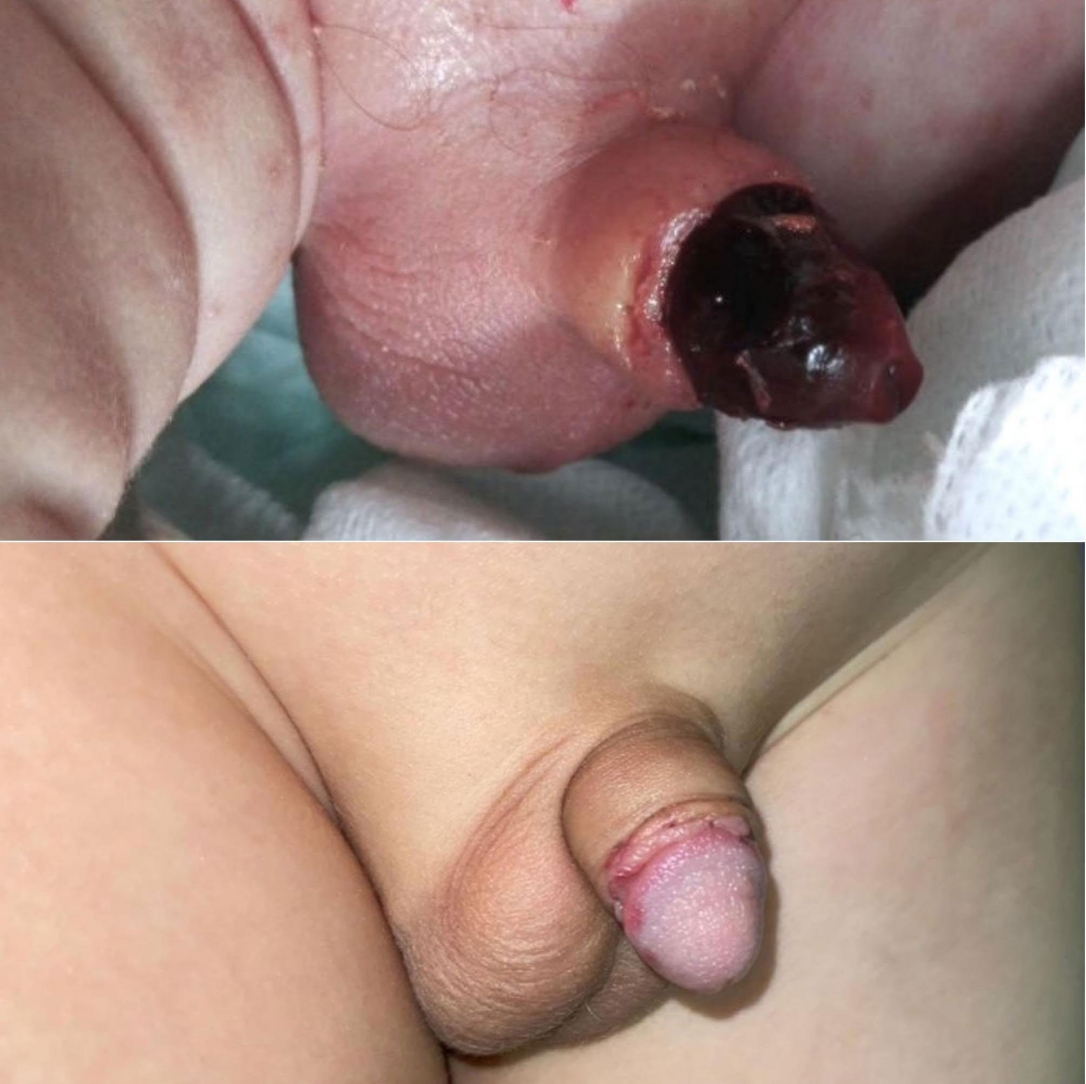
Three months old child with a limited superficial glandular ischemia, and he recovered completely after management with our protocol of HBO, pentoxifylline and nitroglycerin ointment (2%), and he showed a complete recovery

Response of cases to our regimen of HBO, pentoxifylline infusion dressing with nitroglycerin ointment dressing was a complete recovery in 4 cases (17.4%). (Fig. 6)

**Fig. 7:**
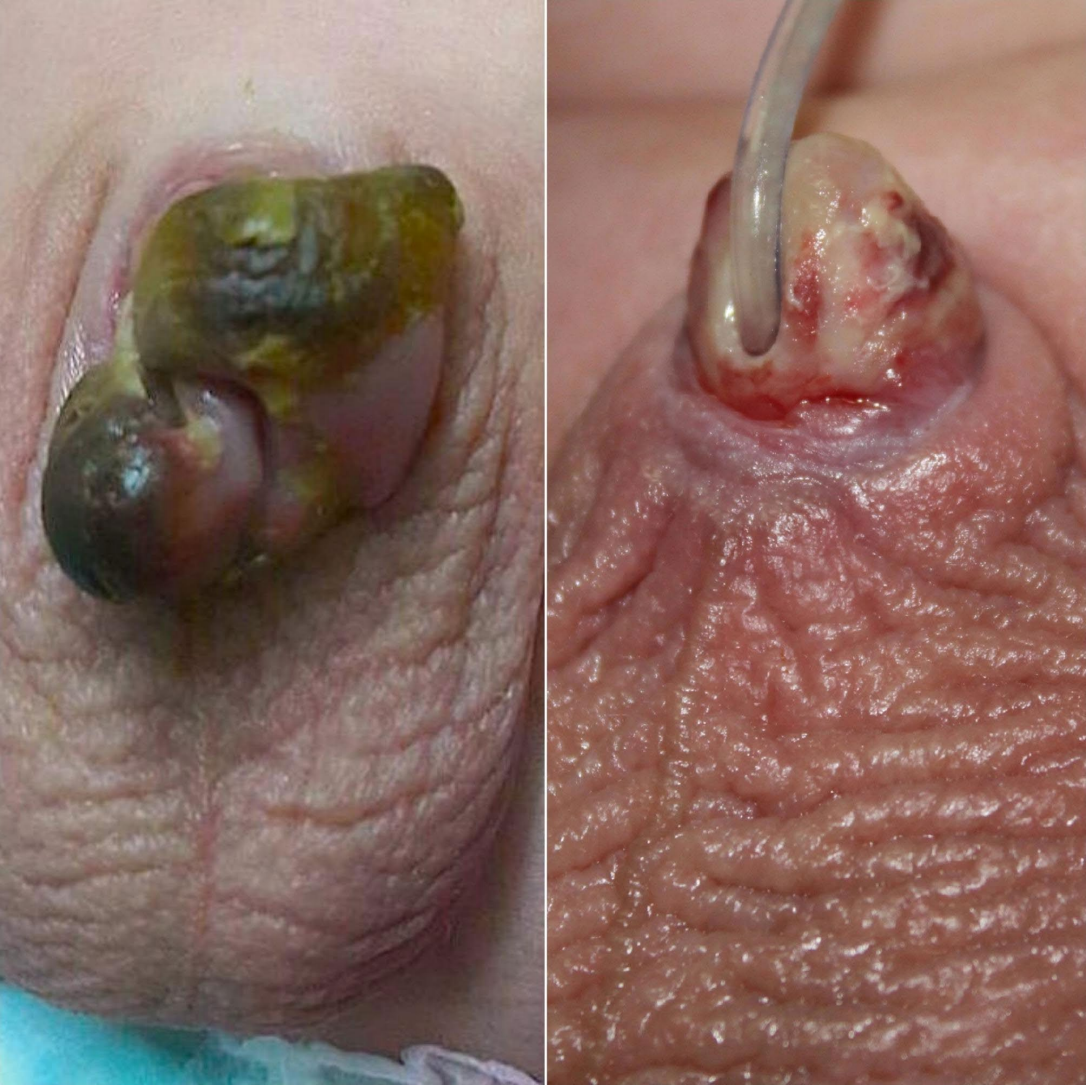
A case of post treatment protocol with a residual iatrogenic distal hypospadias after complete penile ischemic involvement and infection

Glandular and urethral loss: 2 (8.8%). Urethral loss ended with an iatrogenic hypospadias 3 (13%) (Fig. 7). Partial penile loss in 6 (26%). (Fig. 3) Complete penile loss: 2 (8.7%) (Fig. 5); and all those cases were scheduled for further penile reconstruction (13 cases).

Children with iatrogenic hypospadias were repaired by staged reconstructions with buccal mucosal grafting. Patients with complete penile loss developed several episodes of meatal strictures; one of them managed by repeated dilations and the other by suprapubic vesicostomy, and they will have penile reconstruction latter on. Two of the six children who had a partial penile loss were subjected to glanduloplasty with a make use of the buccal mucosa, but others were lost from the long term follow up.

Outcome in relation to the degree of ischemia (superficial or deep) was significant (P = 0.001).

The duration of hospitalization was 10 to 21 days (mean 14 day), and the follow-up period ranged from 6 months to 3 years (mean 18 months).

## Discussion

Penile ischemia is a unique variety of post-circumcision complication that is mainly induced by monopolar diathermy, local anesthetic drugs containing vasoconstrictive agents, and infections [3,12]. Because the dorsal penile artery is a terminal artery, so it is liable to coagulation when one uses this type of electrical monopolar cautery. A study carried out by Tasci et al. in 2020 reported that monopolar diathermy application constitutes the most significant risk factor for necrosis in penile surgeries [3]. If monopolar energy sources are used, the electrical flow can be transferred from the small penile bore and spread to the whole-penis [13]. Thermal damage can result in gangrene and a complete penile loss. (Fig. 5).

In most cases, circumcisions that result in penile necrosis are carried out by junior doctors, general practitioner physicians, or non-medical personnel [13]. These groups of circumcisors are generally not aware of the dangers associated the use of monopolar diathermy or the careless injection of the anesthetic material adjacent to arterial supply and probably the use of local anesthesia with a vasoconstrictor for the penile block. They are also usually incapable of diagnosing penile necrosis early enough; so, they are responsible for the delayed referral of those patients to the appropriate specialists [3]. The delayed transfer of patients is associated with a poor prognosis in this series of patients. Usually, inexperienced physicians manage post-circumcision bleeding by applying a compressive wound dressings for many hours. This act is a technical error and constitutes as a risk factor for glans necrosis [14]. In case of bleeding or vascular injury, minimal bleeding could be stopped safely as recommended by WHO through applying pressure dressings which can stop 90% of bleeding, if this is not amenable; bleeding could be controlled with a careful fine surgical stitches or bipolar diathermy. The low incidence of glans ischemia (4.4%) with plastibell technique is not significant because this technique is not so popular in our locality (10% of the cases). It is proved that the malpractice associated with the use of plastibell rings leads to glans ischemia [2]. The improper use of local anesthesia that contains vasoconstrictors is a major cause of penile ischemia [3]. The etiology of this vascular insult may be vascular spasm at the glans, which is secondary to aggressive surgical manipulations. Also, DPNB may be responsible for dorsal penile artery spasm because of vascular wall irritation or an erroneous injection into the deep dorsal vein of the penis, which leads to venospasm [3&^18^]. Vascular obstruction may occur secondary to the mass effect of the anesthetic solution or hematoma at the site of injection [15]. Any evidence of a possible vascular trauma at the time of the nerve block, dark coloration of the glans penis, or if the child had pain whose severity is disproportionate to physical findings; should draw attention to penile necrosis and mandate deferring the circumcision and close monitoring of penile perfusions [2&^15^].

Post-circumcision surgical site infections are blamed on the pathogenesis of circumcision-related ischemia [14] (43.5%). Wound infection occurs infrequently, due to the premium double blood supply of the penis [4]. It occurs due to the disruption of the skin that acts as a barrier against infection. So, MC, like any other surgical procedure, should be performed under completely aseptic conditions. These infections are usually polymicrobial, either gram-positive or gram-negative, with or without anaerobic organisms. Causative organisms are usually *Staphylococcus aureus*. Actinobacteria normally inhabit the gastrointestinal tract, and it could contaminate circumcision wound due to the dirty environment of the infants’ diapers [4]. According to the culture sensitivity profile, all infected patients in this study responded to antibiotics. Necrotizing fasciitis can result from severe infection. Bliss et al. [7] (1997) reported the occurrence of necrotizing fasciitis in two neonates after routine plastibell circumcision [7]. The symptoms and signs of ischemia are wound erythema, induration, pain, tachycardia, and an elevated leukocyte count [14]. The major goals of our treatment protocol are to provide sufficient blood supply, maximising the possibility for improving the circulation and oxygen delivery to devitalising tissue to minimise further necrosis, and to control tissue infection. Pentoxifylline is responsible for peripheral vasodilation and decreasing the blood viscosity, therefore it increases arterial flow and venous drainage, which may allow for improvement of the impaired tissue vascularity or even revascularization of the ischemic tissues. It can be used safely in neonates, children, and adults [2]. Nitroglycerin dressing is helpful in the wound-healing process by virtue of its topical vasodilator effect. Nitroglycerin could be used in early/superficial cases of penile ischemia to improve local perfusion prior to the commencement of Pentoxifylline and HBOT [9].

Hyperbaric oxygen therapy (HBOT) is a treatment modality which was used during World War II to treat military deep-sea divers and other injured military personnel [16]. HBOT controls the infection and promotes healing, it also potentiates tissue oxygenation by improving angiogenesis via the stimulation of mediators like hypoxia-induced factors and vascular endothelial growth factors and also increasing collagen matrix formation [8&^9^]. It induces the proliferation of fibroblasts by increasing the production of fibroblast growth factor and Transforming Growth Factor [11]. It has a direct toxic effect on anaerobic bacteria by enhancing the production of oxygen free radicals, which oxidize proteins and membrane lipids, destroy DNA, and decrease bacterial metabolism. It improves the functioning of neutrophils by facilitating the oxygen-dependent peroxidase system by which leucocytes kill bacteria and improves the transport of certain antibiotics across the bacterial cell walls [3&^8^]. Absolute contraindications for HBO therapy are in patients complaining of active viral disease or intercurrent pneumothorax [17]. The main complications of HBO are decompression sickness, barotrauma, and arterial gas embolism, but all are very rarely reported. These complications could be also eliminated by avoiding risk factors such as dehydration, middle ear obstruction, otitis media, flying after a hyperbaric dive, and heavy exercise [18]. HBO is useful in some other diseases, as reported by Nehemen et al. in 2020, who used it for crippled hypospadias and other penoscrotal diseases of adults like Fournier gangrene, varicocele-related infertility, penile ischemia caused by injury, and testicular torsion [19]. HBO therapy improved oral autograft taken in 32 cases of hypospadias reoperations patients [19]. Polak et al. (2020) reported a case of an eight-day-old infant with glans amputation during circumcision, the glans was anastomosed over a urinary catheter without vascular anastomosis and showed significant improvement after HBOT [12]. Liu et al. (2019) reported a case of a twenty-six-year-old depressed man who had amputated his penis, testes, and scrotal skin with a knife, a microscopic replantation of the amputated genitals was carried out, and the patient improved with the assistance of HBOT for four weeks [20]. The presented protocol for treatment of the post circumcision penile ischemia results in a good result with superficial degrees of penile ischemia. It minimizes tissue loss in some other patients with deep ischemia. Patients who were diagnosed early and started treatment within the first 24 h had a better prognosis than those who presented after. Infections with anaerobic bacteria are associated with a poor prognosis. Patients with partial or complete penile loss are scheduled individually for further reconstructive surgery according to the degree of tissue loss. It is crucial for the surgeon to detect symptoms of penile ischemia following circumcision with early referral to a specialized and equipped centers for proper evaluation and to start the specific treatment accordingly.

### Study limitations

Limitations of this study are; the limited number of the cases and the sample of the patients is a highly selected group of patients in this study, so, another comparative study or even a double-blind, randomized trial is needed to compare our results with other protocols and treatment modalities before recommending our protocol as a standard of care for post MC penile ischaemia. Also, the degree of penile ischemia was assessed roughly on only clinical bases, a precise investigation is required to measure tissue ischemia objectively. We are unable to determine whether the local anesthetic agent used for DPNB contain a vasoconstrictor or not.

## Conclusion

The protocol of combined HBOT, pentoxifylline infusion and nitroglycerine local ointment is effective in managing patients with post-circumcision penile ischemia. The best results were obtained for a superficial degree of ischemia with early intervention. The use of monopolar diathermy must be prohibited, and roper care for circumcision wounds is essential for the early control of infection.

## Data Availability

All data generated or analysed during this study are included in this published article, other raw materials and relevant data are available upon reasonable request from the corresponding author: ymabfahmy@yahoo.com .

## Abbreviations

MC: Male circumcision
HBOT: Hyperbaric oxygen therapy
RBCs: Red Blood Cells RBCs
DPNB: Dorsal penile nerve block

## References

[1] Morris Brian J, Wamai Richard G, Henebeng Esther B (2016). Estimation of country-specific and global prevalence of male circumcision. Popul Health Metrics.

[2] Mirnia K, Safari A, Saeedi M, Sangsari R (2021). Glans ischemia treatment with pentoxifylline following circumcision in a neonate. J Compr Pediatr.

[3] Tasci AI, Danacioglu YO, Arikan Y, Colakoglu Y, Yapar B, Buyuk Y (2020). Management of post-circumcision necrosis of the penis: the medicolegal aspect. Pediatr Surg Int.

[4] Krill Aaron J, Palmer Lane S, Palmer Jeffrey S (2011). Complications of circumcision. Sci World J.

[5] İnce, Bilsev (2016). Dadacı Mehmet, Altuntaş Zeynep, Bilgen Fatma. Rarely seen complications of circumcision, and their management. Turkish J Urol.

[6] Ölçücü MT, Teke K (2020). Evaluation of short-term postoperative complications according to the Clavien-Dindo classification system in thermocautery-assisted circumcision cases. J Urol Surg.

[7] Bliss David P, Healey Patrick J, Waldhausen John HT (1997). Necrotizing fasciitis after Plastibell circumcision. J Pediatr.

[8] Abdel-Aal Ahmad R, Baky Fahmy Mohamed A, Chapter. 13. Role of Hyperbaric Oxygen Therapy in Male Circumcision Complications in Mohamed A Baky Fahmy; Complications in male circumcision. 2019 Elsevier Inc pp 171–175.

[9] Shou-Hung TYuan-ShengT, Meng E (2004). Lin Teng-Fu, Sun Guang-Huan. Ischemic glans penis after circumcision. Asian J Androl.

[10] Aslan, Adnan (2005). Karagüzel Güngör, Melikoǧlu Mustafa. Severe ischemia of the glans penis following circumcision: a successful treatment via pentoxifylline. Int J Urol.

[11] Bayraktar S, Tanyeri-Bayraktar B (2021). Pentoxifylline in the treatment of neonatal vasospasm and thromboembolism: an observational case series study. J Clin Pharm Ther.

[12] Polak N, Fishelev G, Lang E, Wang Z, Neheman A, Ben Haim Y, Hadanny A, Efrati S. Hyperbaric oxygen as Salvage Therapy for Neonates suffering from critical ischemia of the Glans Penis after Circumcision. Urol 2021 Mar;149: e48–e51. Doi: 10.1016/j.urology.2020.09.006. Epub 2020 Sep 18. PMID: 32956687. 10.1016/j.urology.2020.09.006 32956687

[13] Ceylan K, Burhan K (2007). Yüksel Yılmaz, Şaban Can, Alpaslan Kuş, and Güneş Mustafa. “Severe complications of circumcision: an analysis of 48 cases. J Pediatr Urol.

[14] Pepe Pietro P, Francesco C, Giuseppe P, Michele (2015). Ischemia of the glans penis following circumcision: case report and revision of the literature. Arch Ital Urol Androl.

[15] Zayid Tarek O, Mohamed O, Elbatawy Amr, Zidan Serag M, Hamdy Abdelnaser I, Hany S, Khallad (2020). Dahshan Hazem, Ayad Wael. Two-stage pediatric penile reconstruction after postcircumcision gangrene. J Reconstr Microsurgery Open.

[16] Migliorini Filippo B, Francesco B, Leonardo PA, Benito A, Walter (2018). Acute ischemia of the glans penis after circumcision treated with hyperbaric therapy and pentoxifylline: case report and revision of the literature. Urol Int.

[17] Watdhausen JHT, Holterman MJ, Sawln RS (1996). Surgical implications of necrotizing fasciitis in children with chicken pox. J Pediatr Surg.

[18] Brummelkamp WH, Hogendijk J, Boerema I (1961). Treatment of anaerobic infections (clostridial myositis) by drenching the tissues with oxygen under high atmospheric pressure. Surgery.

[19] Neheman Amos R, Yishai H, Verhovsky Guy B, Nicol S, Warren L, Erez Z, Amnon E, Shai. Hyperbaric oxygen therapy for pediatric “hypospadias cripple”—Evaluating the advantages regarding graft take. Journal of Pediatric Urology 2020; 16(2): 163.e1-163.e7, ISSN 1477–5131. doi: 10.1016/j.jpurol.2020.01.002. 10.1016/j.jpurol.2020.01.002 32171667

[20] Liu XD, Li YF, Wang Q, Zhang Y, Luo Y, Zhou B, Huang ZM, Nie ZL, Li K, Feng QX, Jiang J (2020). Microscopic replantation of completely amputated penis and testes: a case report and literature review. Int Urol Nephrol.

